# IGRA test for TB in COVID-19: role of corticosteroids

**DOI:** 10.5588/ijtld.22.0283

**Published:** 2022-11-01

**Authors:** B. Granozzi, F. Bisognin, M. Tadolini, G. Lombardi, E. Zangoli, D. Salvi, A. Dormi, P. Dal Monte

**Affiliations:** 1 Infectious Diseases Unit, Istituto di Ricovero e Cura a Carattere Scientifico (IRCCS) Azienda Ospedaliero, Universitaria di Bologna, Bologna, Italy; 2 Department of Medical and Surgical Sciences, Alma Mater Studiorum University of Bologna, Bologna, Italy; 3 Microbiology Unit, IRCCS Azienda Ospedaliero, Universitaria di Bologna, Bologna, Italy; 4 Department of Experimental, Diagnostic and Specialty Medicine, Alma Mater Studiorum University of Bologna, Bologna, Italy; 5 Respiratory and Critical care Unit, IRCCS Azienda Ospedaliero, Universitaria di Bologna, Bologna, Italy; 6 Digestive Endoscopy Unit and Gastroenterology, Fondazione Poliambulanza, Brescia, Italy; 7 Digestive Endoscopy Unit, Catholic University of the Sacred Heart, Rome, Italy

Dear Editor,

Although the interaction between TB and COVID-19 is unclear, recent evidence suggests that COVID-19 could worsen the outcome of active pulmonary TB or aggravate respiratory sequelae, especially in settings with high levels of TB infection.^1^–^4^ The immunosuppressive and anti-inflammatory therapies for COVID-19 aim to contain tissue damage induced by the cytokine storm.^5^–^8^ For example, guidelines on the treatment and management of patients with COVID-19 recommend the use of corticosteroids (dexamethasone) in hospitalised patients with severe disease and dexamethasone and other immunomodulatory drugs such as tocilizumab, an interleukin 6 receptor antagonist, in critically ill patients.^9^ Although immunosuppressive therapies for COVID-19 are generally short-lived, the risk of activation of TB infection (TBI) should be kept in mind. Screening for TBI may thus be useful in patients with severe COVID-19, especially in high-resource countries. Among interferon-gamma release assays (IGRAs), the Quanti-FERON-TB Gold Plus (QFT-Plus; Qiagen, Hilden, Germany) test is based on the levels of interferon-gamma (IFN-γ) released by a CD4+ and CD8+ T-cell-mediated immune response after in vitro stimulation of human whole-blood by antigens specific to the *Mycobacterium tuberculosis* complex. Furthermore, the QFT-Plus assay includes a mitogen-based control designed non-specifically to elicit a T-cell response, and thus assess the immunological fitness of the patient. QFT-Plus assay results could therefore be affected by conditions responsible for immune dysregulation. IGRAs have been performed in many hospitalised patients with COVID-19 to rule out TBI before prescribing anti-inflammatory and immunosuppressive treatments, but this has not been systematic. Some studies have reported that the QFT-Plus test had a high and unexpected rate of indeterminate results in COVID-19 patients with severe lung disease.^10^–^12^ The primary aim of this study is to describe the results of the IGRA test among patients with severe COVID-19 who were eligible for immunosuppressive therapies and admitted to a referral hospital in Bologna, Northern Italy. We also assessed the prevalence, clinical impact and factors associated with indeterminate QFT-Plus result in the study population.

We conducted a retrospective, monocentric study enrolling hospitalised adult patients with COVID-19 who underwent QFT-Plus testing between 1 October and 25 November 2020. This study was approved by the Ethic Committee of Area Vasta Emilia Centro (AVEC), Bologna, Italy (Protocol code n. 550/2020/Oss/AOUBo). All patients enrolled had a positive nasopharyngeal swab for SARS CoV-2 using real-time polymerase chain reaction. All subjects with incomplete clinical and laboratory data have been excluded. We collected the following data on each individual: demographics, comorbidities, previous exposure to immunosuppressive therapies, date of onset of COVID-19 symptoms, COVID-19 diagnosis date, date of admission, need of intensive care, steroid or other immunosuppressive therapy administered for COVID-19, laboratory data at admission, date and result of QTF-Plus and outcome (death, survival). The QFT-Plus test was carried out using the chemiluminescence immunoassay method per the manufacturer instructions.^13^ Test results were expressed in quantitative and qualitative terms (positive, negative and indeterminate) in accordance with the manufacturer’s algorithms. Continuous variables were expressed as mean ± standard deviation or median with interquartile range as appropriate, and categorical variables were expressed as percentages. Comparisons between two groups were made using the Mann–Whitney *U*-test for continuous variables, and the χ^2^ or Fisher test for categorical variables. *P* ≤ 0.05 was considered statistically significant. All the analyses were conducted using SPSS v25 (IBM Corp, Armonk, NY, USA).

Of a total of 676 patients hospitalised due to COVID-19 during this period, 268 (39.6%) subjects have been enrolled. The mean age was 68.6 ± 15.0 years; 69.0% were males, 86.6% were born in Italy. Among these, 209 (78.0%) had a determinate QTF-plus result: 199/209 (95.2%) had a negative result and 10/209 (4.7%) were positive; 59/268 had indeterminate test results (22.0%), all of whom showed a lack of response to mitogen control. Study population characteristics according to QFT-Plus results are shown in the Table. The demographic variables, comorbidities, previous immunosuppressive therapy, time between symptoms onset and QFT-Plus and time between admission and QFT-plus testing were not correlated with the indeterminate outcome. A significant correlation (*P* = 0.033) was found between the use of corticosteroids for COVID-19 and an indeterminate QTF-Plus result.

**Table:** Study population characteristics disaggregated by QFT-Plus result

	Total *n*	QFT-Plus indeterminate *n* (%)	QFT-Plus determinate *n* (%)	*P* value	OR (95% CI)
Total	268	59 (22.0)	209 (78.0)		
Sex					
Male	185	39 (21.1)	146 (78.9)	0.582	0.841 (0.455–1.556)
Female	83	20 (24.1)	63 (75.9)		
Age, years, mean ± SD	68.6 ± 15.0	71.0 ± 14.4	67.9 ± 15.2	0.166	
Age groups, years				0.122	1.624 (0.875–3.015)
≤65	105	18 (17.1)	87 (82.9)		
>65	163	41 (25.2)	122 (74.8)		
Origin				0.206	1.881 (0.679–5.074)
Italian	232	54 (23.3)	178 (76.7)		
Foreign-born	36	5 (13.9)	31 (86.1)		
Comorbidities	87	15 (17.2)	72 (82.8)	0.250	0.649 (0.338–1.245)
Haematological disease	14	2 (14.3)	12 (85.7)	0.550	0.769 (0.153–3.858)
Malignancies	6	0 (0)	6 (100)	0.584	0.917 (0.855–0.983)
HIV infection	1	0 (0)	1 (100)	0.828	0.986 (0.959–1.014)
Chronic renal failure	26	4 (15.4)	22 (84.6)	0.516	0.826 (0.237–2.883)
Diabetes	48	9 (18.8)	39 (81.2)	0.898	1.269 (0.409–3.938)
Transplant recipient	4	0 (0)	4 (100)	0.462	0.944 (0.893–0.999)
Other	12	3 (25.0)	9 (75.0)	0.340	1.750 (0.413–7.424)
Chronic immuno-suppressive treatment				0.932	1.046 (0.369–2.964)
Yes	22	5 (22.7)	17 (77.3)		
No	246	54 (22.0)	192 (78.0)		
Steroid treatment before QFT-Plus				0.033	
Yes	217	53 (24.4)	164 (75.6)		
No	51^f^	6 (11.8)	45 (88.2)		
Steroid start	244			0.015	
0–1 days before QFT-Plus	55	10 (18.2)	45 (81.8)		
1.1–2 days before QFT-Plus	67	12 (17.9)	55 (82.1)		
>2 days before QFT-Plus	95	31 (32.6)	64 (67.4)		
After QTF-Plus	27	2 (7.4)	25 (92.6)		
Time between symptoms onset and QFT-Plus, days (range)		8 (6–11)	8 (5–11)	0.460	
Time between admission and QFT-Plus, days (range)		1 (1–3)	1 (1–2)	0.430	
Admission in intensive care unit				0.005	2.775 (1.534–5.019)
Yes	107	35 (32.7)	72 (67.3)		
No	161	24 (14.9)	137 (85.1)		
Outcome				0.016	2.167 (1.143–4.108)
Survival	208	39 (18.7)	169 (81.3)		
Death	60	20 (33.3)	40 (66.7)		
Laboratory data (range)					
Lymphocytes, ×10^9^/L	264	0.64 (0.45–1.04)	1.05 (0.74–1.46)	0.000	
IL-6, pg/ml	230	49.3 (13.1–109.4)	37.4 (17.5–70.4)	0.390	
Ferritin, ng/ml	239	644.5 (351.5–1152.0)	398.0 (222.5–775.0)	0.001	
LDH, U/L	257	383.0 (299.0–458.0)	285.5 (230.2–384.2)	0.000	
PaO_2_/FiO_2_	253	181.5 (121.0–260.5)	249.0 (176.0–321.0)	0.000	
Disease severity				0.015	
PaO_2_/FiO_2_ <100	21	8 (38.1)	13 (61.9)		
PaO_2_/FiO_2_ >100	232	48 (20.7)	184 (79.3)		

* Including 27 cases who started steroid treatment only after performing the QFT-Plus test and 24 cases who did not receive corticosteroid therapy.

QFT = QuantiFERON; OR = odds ratio; CI = confidence interval; SD = standard deviation; IL = interleukin; LDH = lactate dehydrogenase; PaO_2_ = partial pressure of oxygen; FiO_2_ = fractional oxygen.

The longer the duration of corticosteroid therapy before the QTF-Plus, the higher the probability of having an indeterminate result (*P* = 0.015) (Table and Figure). Indeterminate results were also associated with baseline lymphocytes count (*P* = 0.000), ferritin (*P* = 0.001), lactate dehydrogenase (LDH, *P* = 0.000), severity of COVID-19 disease (expressed in terms of ratio of arterial oxygen partial pressure (PaO_2_ in mmHg) to fractional inspired oxygen [PaO_2_/FiO_2_] < 100, *P* = 0.015), need of intensive care (*P* = 0.005) and negative outcome (death, *P* = 0.016).

Our study confirms a high rate of indeterminate QTF-Plus outcome in COVID-19 patients (22%). In healthy populations and in pre-pandemic conditions, indeterminate results occurred on average in approximately 2–11% of the tests in similar low-burden settings.^14^ Other studies have reported even higher indeterminate rates among COVID-19 patients, up to 40.3%.^10^–^12^ In our experience, immune dysregulation due to the coexistence of immunosuppressive conditions (such as diabetes or haematological diseases or malignancies) do not seem to influence the QFT-Plus result. Similarly, in this study, the chronic use of corticosteroid or other immunosuppressant therapies was not associated with a higher risk of indeterminate QFT-Plus results than in the study population (22.7% vs. 22.0%, respectively). Instead, a significant correlation with the use of corticosteroid therapy due to COVID-19 disease emerged, especially when steroid use was started ≥1 day before the QFT-Plus test was performed. To note, indeterminate result occurred also among those who did not receive corticosteroid therapy during hospitalisation (4/24, 16.7%) or those who started steroid treatment only after the QFT-Plus test (2/27, 7.4%). Hence, we can hypothesise that COVID-19 and its pathogenetic process play a direct role in reducing response to mitogen and influencing QFT-Plus outcome. As SARS-CoV-2 leads to a decrease in the number of lymphocytes, the level of IFN-γ released by CD4+ T-cells is anticipated to be lower in severe cases.^15^ It was also found that patients with indeterminate outcomes had more severe hypoxemia than patients with determinate results, with considerably lower PaO_2_/FiO_2_ values, used as proxy for the severity of respiratory failure. As reported by other studies, indeterminate results were correlated with the need for intensive care and death, which supports the hypothesis that disease severity and test outcome were correlated.

**Figure 1:**
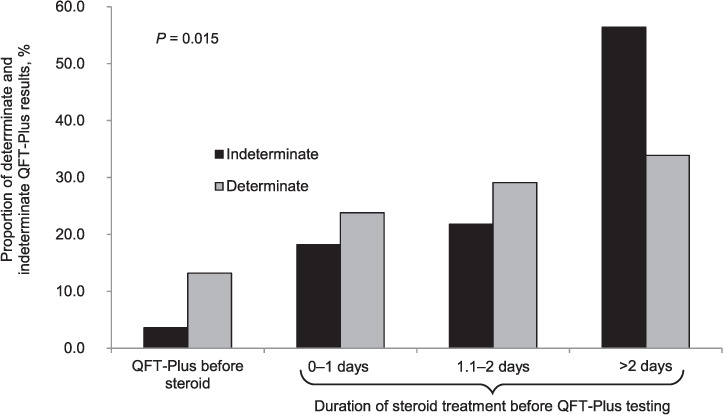
QFT-Plus results according to duration of steroid therapy. QFT = QuantiFERON.

In conclusion, our data confirm the occurrence of high rates of QTF-Plus-indeterminate results among hospitalised COVID-19 patients, and suggest that SARS-Cov-2, combined with the use of corticosteroid drugs, are main risk factors. Our findings cast doubt on the usefulness of IGRA tests in immunosuppressive pre-treatment screening of COVID-19 patients. However, indeterminate QTF-Plus results may help predict the severity and mortality of COVID-19 disease. Other studies are needed to confirm these hypotheses.
